# Syndromic and Monogenic Obesity: New Opportunities Due to Genetic-Based Pharmacological Treatment

**DOI:** 10.3390/children11020153

**Published:** 2024-01-25

**Authors:** Kallirhoe Kalinderi, Vasiliki Goula, Evdoxia Sapountzi, Vasiliki Rengina Tsinopoulou, Liana Fidani

**Affiliations:** 1 Laboratory of Medical Biology-Genetics, School of Medicine, Faculty of Health Sciences, Aristotle University of Thessaloniki, 54124 Thessaloniki, Greece; kkalinde@auth.gr,; 2 School of Medicine, Faculty of Health Sciences, Aristotle University of Thessaloniki, 54124 Thessaloniki, Greece; vasilikig@auth.gr; 3 Second Department of Pediatrics, School of Medicine, Faculty of Health Sciences, AHEPA University Hospital, Aristotle University of Thessaloniki, 54636 Thessaloniki, Greece; sevdoxia@auth.gr (E.S.); vitsinop@auth.gr (V.R.T.)

**Keywords:** genetic obesity, monogenic obesity, syndromic obesity, Prader–Willi syndrome, Bardet–Biedl syndrome, melanocortin 4 receptor, congenital leptin deficiency, setmelanotide, GLP-1 R agonists, semaglutide

## Abstract

Obesity is a significant health problem with a continuously increasing prevalence among children and adolescents that has become a modern pandemic during the last decades. Nowadays, the genetic contribution to obesity is well-established. For this narrative review article, we searched PubMed and Scopus databases for peer-reviewed research, review articles, and meta-analyses regarding the genetics of obesity and current pharmacological treatment, published in the English language with no time restrictions. We also screened the references of the selected articles for possible additional articles in order to include most of the key recent evidence. Our research was conducted between December 2022 and December 2023. We used the terms “obesity”, “genetics”, “monogenic”, “syndromic”, “drugs”, “autosomal dominant”, “autosomal recessive”, “leptin-melanocortin pathway”, and “children” in different combinations. Recognizing the genetic background in obesity can enhance the effectiveness of treatment. During the last years, intense research in the field of obesity treatment has increased the number of available drugs. This review analyzes the main categories of syndromic and monogenic obesity discussing current data on genetic-based pharmacological treatment of genetic obesity and highlighting the necessity that cases of genetic obesity should follow specific, pharmacological treatment based on their genetic background.

## 1. Introduction

Obesity is a metabolic disorder in which energy balance is dysregulated, leading to weight gain. It is a major medical problem beginning from childhood and seems to have an epidemic size as the World Health Organization has estimated the number of children < 5 years old who are obese or overweight to be ~39 million [1]. Children are considered overweight when their BMI is between the 85th and 95th percentile for their age and gender, obese with a BMI ≥ 95th percentile, and extremely obese with a BMI ≥ 120% of the 95th percentile [2]. According to the World Obesity Atlas 2023 report, 38% of the global population is currently either overweight or obese. In a recent WHO report based on data collected from 411,000 children aged 6–9 years from 33 countries of the WHO European Region, 29% of children aged 7–9 years were found to live as overweight or obese, thus almost 1 in 3 children were overweight or obese. According to the WHO organization, Greece ranks first in the European Union in childhood obesity, with 20.6% of children aged 4–6 years being obese or overweight with this percentage increasing to 38.5% and 41.2% in children aged 6–10 and 10–12 years, respectively [1]. Moreover, 37.9% of Greek adults are overweight and 24.9% are obese. Importantly, a large percentage of obese children become obese adults with many comorbidities such as type 2 diabetes (T2D), hypertension, and cardiovascular diseases (CVD) and an increased risk of early death [2]. Thus, early detection and the prompt use of strategies against obesity are of crucial importance.

Previous genetic studies have shown that there is a genetic factor in the development of obesity [3]. According to the genetic involvement, obesity is classified into (i) “common polygenic obesity,” which results from the interplay of genetic and environmental factors. Hundreds of polymorphisms that each have a small effect contribute to common obesity. With the advent of genome-wide association studies, multiple genes and loci such as *FTO*, *TMEM18*, *CADM1*, *CADM2*, and *NEGR1* have been associated with increased susceptibility to common, polygenic obesity; (ii) “syndromic obesity”, which except for obesity is characterized by neurodevelopmental delay or dysmorphic features such as Prader–Willi and Bardet–Biedl syndrome and “monogenic obesity”, which is typically rare, early-onset, severe, inherited in a Mendelian pattern, and results from single gene mutations with large effects. This classification has been reconsidered, as cases with monogenic obesity may be accompanied by neurodevelopmental characteristics or psychiatric conditions that are usually seen in syndromic cases. Thus, obesity syndromes can be evaluated separately from polygenic obesity [4]. In this review “syndromic” and “monogenic” forms of obesity are discussed focusing on the most well-known representatives of each category.

## 2. Syndromic Obesity

Syndromic obesity refers to obesity that is associated with intellectual disability, dysmorphic features, or abnormalities affecting different organs and systems, with low frequency, high variability, and a Mendelian pattern of inheritance [5].

### 2.1. Prader–Willi Syndrome (PWS)

PWS is an imprinting disorder with an incidence of ~1/15,000–20,000 [6]. Babies with this syndrome are born “floppy”, with hypotonia and up-slanted palpebral fissures, and feed poorly. However, over the first two years, the infant slowly develops hyperphagia and shows developmental and cognitive delays and behavioral problems. PWS obesity is age-dependent, being ~40% and ~85% in children/adolescents and in adulthood, respectively [7]. Multiple endocrine abnormalities such as growth hormone deficiency, hypothyroidism, hypogonadism, and leptin resistance are also seen in PW patients, due to hypothalamus–pituitary–gonadal axis dysregulation. Moreover, PW patients are characterized by an increased incidence of metabolic complications, such as CVD, T2D, and hypertension [6]. The basic genetic types of PW are due to paternal 15q11.2–q13 deletion present in ~70% of PWS cases, maternal uniparental disomy 15 present in ~30% of PWS cases, and imprinting defects in about ~3%. [6].

### 2.2. Bardet–Biedl Syndrome (BBS)

BBS is a clinically and genetically heterogeneous, autosomal recessive syndrome with a prevalence of about 1/125,000 [8,9], affecting multiple organs in the body. The core clinical characteristics of BBS are severe visual impairment, deformities in extremities, central obesity, mental retardation, renal dysfunction, and male hypogonadism. Obesity in BBS patients involves the dysregulation of the hypothalamic leptin–melanocortin signaling pathway. Commonly associated secondary features of BBS include liver fibrosis, T2D, short stature, speech problems, and neurodevelopmental delay [10].

BBS is a disorder of genetic heterogeneity. Currently, 19 *BBS* genes have been described. *BBS1* and *BBS10* are the most highly mutated genes, reaching percentages of 70–80% in certain Caucasian and ~40–50% in patients from Northern Europe [11]. Most commonly, BBS follows an autosomal recessive mode of inheritance but also ”triallelic inheritance” has been observed [12]. Genetic modifiers may also determine clinical variability in BBS patients.

### 2.3. Pseudohypoparathyroidism (PHP) Type 1a

Pseudohypoparathyroidism (PHP) is a rare disorder characterized by low levels of calcium, high levels of phosphorus, elevated PTH levels in the blood, and parathyroid hormone (PTH) resistance [13]. It is divided into three categories, PHP-1, PHP-2, and pseudopseudohypoparathyroidism (PPHP). PHP-1 has three subtypes: 1a, 1b, and 1c. PHP type 1a and 1c display Albright’s hereditary osteodystrophy (AHO) features, including obesity. Mutations in the *GNAS* on chromosome 20q13.2–13.3 have been recognized due to spontaneous mutation or inherited in an autosomal dominant manner. PHP 1b has no AHO characteristics and is restricted only to the kidney. Recently, *GNAS* and *STX16* deletions were associated with PHP 1b [14]. PHP-2 patients have PTH resistance but with no AHO features. Regarding, PPHP patients, they inherit the mutation from the father and GSa is maternally expressed. In PHP 1a, obesity is associated with alterations in the MC4R pathway [15].

### 2.4. Alström Syndrome (ALMS)

ALMS is a rare autosomal recessive syndrome caused by *ALMS* mutations located on chromosome 2. Current incidence is unknown with estimates ranging from 1 in 500,000 to 1 in 1,000,000 [16,17]. ALMS affects multiple organs and is characterized by clinical heterogeneity. ALMS patients usually suffer from vision and hearing loss, central obesity, T2D, dilated cardiomyopathy or congestive heart failure, infertility, acanthosis nigricans, hypothyroidism, and short stature. Fibrosis in the kidneys, liver, and lungs can lead to its dysfunction. Delayed learning skills are also common [18].

### 2.5. 16p11.2 Deletion Syndrome

16p11.2 deletion syndrome is a microdeletion syndrome with an estimated prevalence of 1–5/10,000. It is usually caused by a small deletion of chromosome 16 at position p11.2. De novo deletions are frequent [19]. The main clinical characteristics of this syndrome are neurodevelopmental delay, intellectual disability, and autism [20]. A strong association between 16p11.2 microdeletion and obesity has been reported [21]. Among the genes involved in this microdeletion syndrome is *SH2B1*, which participates in the leptin–melanocortin pathway. Some other physical abnormalities such as finger clinodactyly and syndactyly, craniofacial or dermatological abnormalities, anxiety disorders, or reduced fertility may also be present. The size of the lost chromosome region actually determines the severity of the syndrome [19].

### 2.6. WAGR Syndrome

WAGR is a syndrome with a prevalence of 1/500,000, which is caused by a deletion of chromosome 11p13 that encompasses the *WT1* and *PAX6* genes [22]. It is an autosomal dominant syndrome that has a susceptibility to Wilms tumor, absence of the iris, genital and urinary abnormalities, and developmental delay. A specific phenotype of WAGR, WAGRO includes obesity, too [23]. Brain-derived neurotrophic factor (*BDNF*) gene haploinsufficiency has been implicated in childhood-onset obesity, intellectual disability, and autism [24]. In a study of 33 patients with WAGR, it was observed that patients with *BDNF* haploinsufficiency had significantly higher BMIs and this was directly associated with childhood-onset obesity [25].

### 2.7. Smith–Magenis Syndrome (SMS)

SMS mainly occurs by a small deletion of chromosome 17p11.2 [26]. Mutations in *RAI1* leading to its haploinsufficiency are more rarely found [27]. SMS is a developmental disorder accompanied by intellectual disability, dysmorphic characteristics, and sleep and behavioral problems. Early in their lives, the majority of patients with SMS become overweight/obese [26].

### 2.8. Cohen Syndrome (CS)

CS is caused by mutations in the vacuolar protein sorting 13 homolog B (*VPS13B*) gene located on chromosome 8q22.2. The so-called “brain-obesity-eye-bone” syndrome is an autosomal recessive disease, with developmental delay, intellectual disability, hypotonia, and specific facial characteristics [28]. Currently, >200 *VPS13B* mutations have been detected in ~200 patients with CS of various ethnicities [29,30]. VPS13B is a transmembrane protein with functional and structural role in the Golgi apparatus [31].

### 2.9. MYT1L-Variants Syndrome

Mutations in the myelin transcription factor 1-like (*MYT1L*) gene have been associated with obesity following an autosomal dominant mode of inheritance, which is mainly accompanied by developmental and behavioral problems, intellectual disability, and epilepsy. MYT1L belongs to the myelin transcription factor 1 (MYT1) family and in humans is only expressed in the brain. Mutations in the *MYT1L* affect the expression of genes that have a crucial role in the neurodevelopment and function of the hypothalamus [32].

### 2.10. Börjeson–Forssman–Lehmann Syndrome (BFLS)

BFLS is an X-linked recessive genetic disease caused by mutations in *PHF6* [33]. It is accompanied by intellectual disability, growth defects, hypogonadism, seizures, and specific facial characteristics. Obesity emerging in late childhood is found in ~75% of BFLS patients who also can have microcephaly (6%) or macrocephaly (15%) [34].

### 2.11. Carpenter Syndrome (CRPT1)

CRPT1 is an autosomal recessive disease caused by homozygous or compound heterozygous loss-of-function mutations in *RAB23*. It is characterized by craniosynostosis, polysyndactyly, obesity mental retardation, hypogonadism, and lateralization defects [35]. *RAB23* encodes a small GTPase of the Ras superfamily and participates in membrane trafficking and vesicular transport, exerting a crucial role in the Sonic hedgehog signaling pathway [36].

### 2.12. Down Syndrome (DS)

DS also known as trisomy 21 occurs in about 1:600–700 newborns. The main causes of the extra copy of chromosome 21 are chromosomal non-disjunction, Robertsonian translocations, and mosaicism. Patients with DS are more often obese probably due to increased levels of leptin, decreased activity, bad eating habits, and abnormal metabolism. Interestingly, in a previous study, the possibility of patients with DS being overweight or obese compared to individuals without developmental disabilities was significantly increased [37,38].

### 2.13. Kallmann Syndrome (KS)

KS is characterized by hypogonadotropic hypogonadism and anosmia. Multiple genes have been implicated in the pathogenesis of KS including *KAL1, FGFR1, FGF8, PROKR2*, and *PROK2*. KS is mainly an X-linked recessive disorder but cases with autosomal recessive or dominant patterns of inheritance have been described [39]. Unilateral renal agenesis, cleft palate, abnormal eye movements, hearing loss, as well as obesity are also seen in these patients.

## 3. Monogenic Obesity

Monogenic obesity follows a Mendelian pattern of inheritance and patients are usually characterized by severe obesity due to mutations in a specific gene. Mutations in genes implicated in the leptin–melanocortin pathway have been mostly associated with monogenic obesity. In this pathway, leptin—an adipose tissue hormone—binds to the hypothalamic leptin receptor, stimulating the production of proopiomelanocortin (POMC). Proprotein convertase subtilisin/kexin type 1 (PCSK1) cleaves POMC into melanocortin ligands, such as α- and β-melanocyte–stimulating hormone, expediting binding and activation of the melanocortin-4 receptor (MC4R), thus reducing food intake and increasing energy consumption. BDNF has also a regulatory role in this pathway [5].

### 3.1. Congenital Leptin Deficiency

Leptin is mainly produced by adipose tissue and has a crucial role in energy metabolism. More specifically, leptin acts in the hypothalamus activating anorexigenic POMC, as well as cocaine- and amphetamine-related transcript preprotein (CARTPT) derivatives that control food consumption. Leptin has also an inhibitory action on the orexigenic peptides neuropeptide Y (NPY) and Agouti-related protein (AGRP), which increase food consumption. When leptin binds to its receptor, a cascade of signals is triggered that can possibly lead to leptin resistance. Serum leptin levels are usually undetectable and clinically heterozygous patients are characterized by early-onset obesity, whereas in a homozygous state, hypogonadotropic hypogonadism and hypothyroidism can also be seen [40,41].

### 3.2. Congenital Leptin Receptor Deficiency

Leptin receptors are present mainly in the hypothalamus but also in other organs, thus, leptin can exert its role in multiple different ways. The *LEPR* gene encodes for six different isoforms (LEPR a–f), among which the LEPR-b receptor has been mostly associated with severe forms of monogenic obesity [42]. Both homozygous or compound heterozygous *LEPR* mutations can lead to leptin receptor deficiency inherited in an autosomal recessive manner. Serum leptin levels are usually increased and severe obesity is prominent in these patients [43].

### 3.3. POMC Deficiency

POMC-derived peptides, such as α-MSH, are produced in the arcuatus nucleus and act via binding to the MC4R, suppressing appetite and food intake, thus having a fundamental role in weight regulation. Patients with *POMC* deficiency are characterized by severe early-onset obesity and follow an autosomal recessive mode of inheritance. Among clinical symptoms are polyphagia, pigmentary abnormalities, and adrenal insufficiency [44].

### 3.4. PCSK1 Deficiency

PCSK1 can activate by cleaving many precursor molecules such as POMC, proinsulin, proglucagon, and proghrelin, which are important for energy metabolism [45]. Individuals with *PCSK1* mutations show severe obesity, polyphagia, diarrhea, hypoglycemia, and other symptoms of hormonal imbalance [46,47]). Biallelic *PCSK1* mutations are associated with impaired prohormone processing [46,47].

### 3.5. MC4R Deficiency

MC4R, also expressed in the hypothalamus, has a crucial effect on energy homeostasis and food consumption. Clinically, mutation carriers are characterized by severe obesity, severe hyperinsulinemia, increased lean body mass, and linear growth, whereas *MC4R* homozygous patients—although rarely identified—are more severely affected [45].

### 3.6. SH2B1 Deficiency

Src homology 2B1 (SH2B1) protein binds to a number of tyrosine kinases, such as Janus kinase 2 (JAK2) and the insulin receptor. SH2B1 is abundantly expressed in the brain and in various tissues including the adipose tissue. SH2B1 participates in the leptin-mediated signal pathway regulating body weight, insulin sensitivity, and glucose metabolism. Individuals with *SH2B1* mutations show obesity, polyphagia, severe insulin resistance, as well as maladaptive behaviors, and follow an autosomal recessive mode of inheritance [48].

### 3.7. CPE Deficiency

Carboxypeptidase E (CPE) catalyzes the activation of hormone precursors to active molecules [49]. CPE is highly expressed in the central nervous system as well as in other tissues including adipose tissue. In 2015, a truncating pathogenic *CPE* mutation was detected in a female patient suffering from severe obesity, hyperglycemia, intellectual disability, and hypogonadotropic hypogonadism [50] and, recently, a new syndrome Blakemore–Durmaz–Vasileiou was described with homozygous *CPE* mutations and similar characteristics plus central hypothyroidism [51].

### 3.8. SRC1 Deficiency

Steroid receptor coactivator-1 (SRC1) affects the expression of many target genes [52]. In a previous large exome-sequencing study of patients with severe obesity, rare heterozygous variants in *SRC1* were detected and were associated with the dysregulation of POMC expression [53,54]. Importantly, *SRC-1* deletion also caused obesity in mice [55]. In a recent study, it was shown that in the hypothalamus, SRC-1 interacts with phosphorylated signal transducer and activator of transcription-3 (STAT3), affecting POMC expression [56]. Patients with *SRC1* mutations and severe obesity have been enrolled in phase 2 clinical trials of setmelanotide, an MC4R agonist, approved for obese patients with *POMC* or *LEPR* mutations [54].

## 4. Genetic-Based Pharmacological Treatment of Obesity

Obesity has become a major health problem worldwide due to its increasing prevalence and comorbidities. About 20% of the children and adolescents in the United States suffer from obesity, nearly 6% of them have severe obesity, and ~7% of severe pediatric obesity has a genetic background [57,58] with almost 3% having *LEPR* mutations [59] and 3–6% *MC4R* mutations [60]. It is well-known that children suffering from obesity are at increased risk for several medical health problems, thus, early diagnosis and intervention are of fundamental importance; however, the available pharmacological armamentarium is currently restricted [61]. Moreover, identifying a child with polyphagia based on a genetic background can be of crucial importance for treatment. Lately, intense research has emerged focusing especially on genetic-based pharmacological treatment of obesity with quite enthusiastic results, which have been accompanied by FDA approval of new agents, eventually increasing the available armamentarium for the pharmacological treatment of genetic obesity (Table 1).

In 1999, leptin deficiency—diagnosed in homozygous LEP gene mutation carriers with severe obesity—was successfully treated with leptin administration [62]. Indeed, subcutaneous injection of human recombinant leptin (metreleptin) is notably beneficial for patients with leptin deficiency, leading to improved food control and weight reduction, as well as improvement of metabolic and endocrine dysregulations [63]. Reported side effects include the production of antibodies against leptin and an increased susceptibility to lymphomas [62].

In 2016, a breakthrough regarding the drug therapy of genetic obesity was made introducing setmelanotide, a new MC4R agonist [64,65]. Initially, in a phase II trial, in two *POMC* homozygous patients, setmelanotide resulted in better food control and considerable weight improvement [64]. In 2020, in a phase III trial, 80% of *POMC*-deficient patients and 45% of *LEPR*-deficient patients had ~10% weight loss at ~1 year. The adverse events of setmelanotide were hyperpigmentation, nausea, vomiting, and injection site reactions [65]. In 2020, setmelanotide was approved by the US Food and Drug Administration (FDA) for chronic weight management in obese adult and pediatric patients ≥ 6 years old with *POMC, PCSK1*, or *LEPR* receptor deficiency [66]. In 2022, a long-term evaluation of the two initial *POMC*-deficient obese patients who were administered setmelanotide for 7 years verified the sustained decrease in BMI and hunger and the absence of serious side effects, apart from hyperpigmentation [67]. Collet et al. also evaluated setmelanotide in patients with *MC4R* deficiency. In this study, the effectiveness of setmelanotide was closely related to the type of *MC4R* mutation [68]. Semelanotide has also been examined in obese BBS patients and obese patients with Alström syndrome. In a phase II study, 10 BBS patients ≥ 12 years old were administered setmelanotide once/day for 1 year, with significant weight loss (16.3%) and reduction in polyphagia [69]. In a phase III trial, setmelanotide was tested in 38 BBS patients and 34% of patients had a weight loss > 10% after 52 weeks of drug administration, without serious side effects [70]. Moreover, in a recent Phase 3 trial after 1 year of use of setmelanotide, adults and children with BBS syndrome reported a better quality of life [71]. In 2022, setmelanotide was also approved by the FDA for the treatment of obesity and binge-eating disorders in > 6 years of age BBS patients. The results of ongoing clinical trials for the use of setmelanotide in patients having mutations in multiple genes connected with the MC4R pathway and in patients with deletions on chromosome 16p11.2 are also awaited with a lot of interest [72].

GLP-1R agonists may also be a novel alternative drug for patients with genetic obesity [73]. GLP-1R is expressed in the central nervous system and peripherally, such as in the β-cells of the pancreas [73]. The use of semaglutide, a long-acting GLP-1R analog, once weekly in patients with obesity has been associated with ≥ 10% weight loss after 52 weeks of drug administration [74,75]. The use of 3 mg liraglutide/day, another long-acting GLP-1R analog, in patients with MC4R variants or PWS for 16 weeks has also been tested, leading to ~6% weight loss in both MC4R-variant carriers and non-carriers, indicating that liraglutide has an appetite-reducing effect and acts independently of the MC4R pathway [76]. In another study, 3 mg liraglutide/day was administered for 16 weeks in a woman with homozygous pathogenic *MC4R* mutation, morbid obesity, and T2D, resulting in a decrease in body weight of ~10 kg, similar to weight loss in heterozygous *MC4R* mutation carriers and common obesity; thus, it can act independently of the *MC4R* mutation status [76,77]. Currently, liraglutide and semaglutide have been approved for obesity treatment. Moreover, tirzepatide, a novel agonist that acts at both the gastric inhibitory polypeptide receptor (GIPR) and GLP-1R, has been documented to lower food consumption as well as affect energy metabolism [78]. GIPRs are found in the central nervous system and in adipose tissue [79]. Interestingly, in a phase III clinical trial, tirzepatide was administered in obese individuals and led to significant weight loss [80,81]. Recently, tirzepatide also received FDA approval for obesity treatment. GLP-1/GIP/glucagon receptor triple agonists are also examined in obesity [82] and many other novel molecules and combinations of agonists are still in development and tested, with promising results.

## 5. Other Current and Promising Therapeutic Approaches

**Figure 1:**
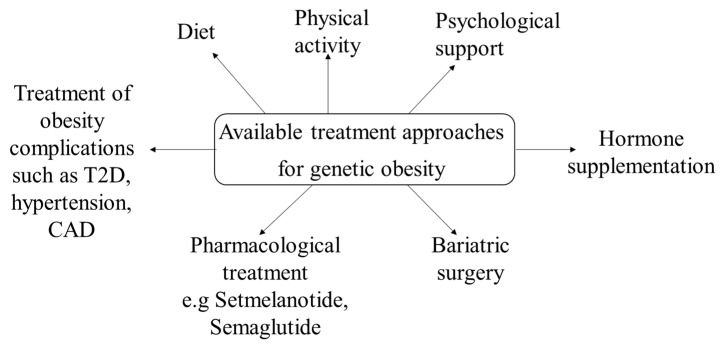
Available treatment approaches for genetic obesity.

Except for the genetic-based pharmacological treatment of obesity—which is mainly discussed in this review article and currently applies mostly in monogenic obesity and in BBS syndrome—as well, there are also other current therapeutic approaches that should be considered in genetic obesity (Figure 1).

In patients with genetic obesity restriction of food consumption, psychological and behavioral support, increased physical activity, and reduced carbohydrate consumption are generally recommended. Possible obesity complications and comorbidities also require appropriate treatment. Importantly, these measures should be initiated as soon as possible during childhood [3]. For instance, in BBS and AS patients and especially in PWS patients, restriction of food intake and a routine regarding the timing of meals is greatly beneficial [83,84]. Decreased carbohydrate intake and increased dietary fiber intake are also recommended in PWS patients [85]. The possible benefits of probiotics in these patients are under investigation. In addition, GH therapy has been approved for the growth development of PWS children and is used after the establishment of the PWS diagnosis [86]. The results of the use of GLP-1 receptor agonists like exenatide, liraglutide, and semaglutide in PWS are currently inconsistent, and additional studies are needed in order for more definite conclusions to be drawn [87,88,89]. Oxytocin and carbetocin, a synthetic analog of oxytocin, have also been administered in PWS patients. In an RCT with 119 PWS patients, those who were given 3.2 mg of carbetocin displayed substantial improvements regarding hyperphagia [90]. Diazoxide choline-controlled release, an extended-release form of diazoxide choline, was also examined in a phase III PWS RCT study and was associated with significantly reduced fat mass [91]. Moreover, a methionine aminopeptidase-2 inhibitor, belanorib, was examined in a phase III PWS trial, with improvement in hyperphagia and weight loss; however, the study was discontinued due to the high incidence of thrombotic events [92]. A combination of tesofensine and metoprolol has also been used in a study including 21 adults with hypothalamic obesity, with a mean weight change of −6.3% in the patient group [93]. A few studies have also examined phentermine/topiramate and bupropion/naltrexone in PWS patients with positive results, however, after treatment discontinuation, weight regain was observed [93,94]. Regarding bariatric surgery, in cases with syndromic obesity, it should be generally considered with caution, as severe behavioral problems, compulsive food behavior, and developmental delays can lead to worse results [3]. Interestingly, in a recent systematic review of metabolic and bariatric surgery in PWS a significant decrease in BMI was observed with promising long-term outcomes [95]. Regarding monogenic obesity, the long-term outcomes of bariatric surgery are questionable, and considering the increasing availability and effectiveness of pharmacological agents, bariatric surgery should be avoided as long as possible [3].

Other alternative promising strategies to counteract obesity include the use of human-induced pluripotent stem cells (hiPSCs). hiPSCs can be differentiated into multiple cell types in vitro; thus, hiPSCs are a promising therapeutic approach for obese patients as they can provide an unlimited amount of brown adipose progenitor cells [96], which in contrast to white adipocyte cells, can dissipate stored energy. With the iPSC methodology, brown adipose progenitor cells can be used for both cell transplantation as well as for anti-obesity drug discovery. In vitro representations of human genetic conditions can also improve the examination of the molecular and cellular impact of certain genetic mutations in energy homeostasis pathways [97,98,99,100,101,102].

Targeted genome editing mediated by clustered, regularly interspaced, short palindromic repeat (CRISPR)/CRISPR-associated nuclease 9 (Cas9) technology has become a robust alternative to examine gene functions and treat genetic disorders, including genetic obesity. Interestingly, in a recent study, the mutated leptin gene in ob/ob mice was edited using CRISPR/Cas9. The ob/ob mice displayed a correction of 1.67% of leptin alleles, which was sufficient to restore the production and function of leptin [103]. Moreover, regulating the expression of obesity-causing genes, like MC4R, by using the CRISPRi technique gene expression has also been suggested to be useful in genetic obesity therapy [104].

The exploitation of small extracellular vesicles (sEVs) In obesity is also promising. sEVs are small cellular vesicles that can deliver specific molecules including lipids, proteins, mRNAs, and noncoding RNAs extracellularly to target cells, affecting their function. Interestingly, recently regulation of hypothalamic AMPK using sEV methodology in obese mouse models resulted in significant weight loss [105].

## 6. Conclusions

Obesity is a modern pandemic, with the WHO recently reporting that almost 1 in 3 children are overweight or obese and, among the European Union, Greece is first in childhood obesity. Importantly, a large percentage of obese children become obese adults with many comorbidities such as type 2 diabetes (T2D), hypertension, cardiovascular diseases (CVD), and an increased risk of early death [2]. This review highlights the importance of genetics in obesity, discussing cases of syndromic obesity such as Prader–Willi and Bardet–Biedl and of common monogenic obesity that are mainly characterized by mutations in the leptin–melanocortin pathway. This review also emphasizes the fact that, in patients with genetic obesity, general measures such as restriction of food consumption, psychological and behavioral support, increased physical activity, and reduced carbohydrate consumption should be initiated as soon as possible during childhood but also focuses on current progress that has been recently made in terms of pharmacological treatment. The MC4R agonist, setmelanotide, should be administered in patients with *POMC, PCSK1*, or *LEPR* deficiency and BBS, and the GLP-1 receptor agonists, semaglutide and liraglutide are also novel pharmacological agents that have been FDA approved for the treatment of childhood obesity. The importance of this review is that it contributes to the awareness of healthcare professionals that identifying a child with polyphagia based on a genetic background can be of crucial importance for its treatment and enhance knowledge on the available armamentarium for the pharmacological treatment of genetic obesity. Alternative strategies against obesity such as hiPSCs, CRISPR technology, and the use of sEVs are also promising future methodologies. In the meanwhile, future long-term studies in an increased number of patients are recommended and awaited to increase the list of available drugs that target certain genetic types of obesity. Combinations of anti-obesity drugs also warrant further study in patients with genetic obesity, aiming to facilitate genetic-based individualized treatment.

## Figures and Tables

**Table 1:** New pharmacological agents approved for genetic obesity.

	Drug Class	Dose	Route	Approved for	Most Common Side Effects
**Metreleptin**	Recombinant analog of leptin	0.03 mg/kg	subcutaneous	LEP deficiency	production of anti-leptin antibodies, increased risk of lymphomas
**Setmelanotide**	MC4R agonist	Max 3 mg	subcutaneous	POMC deficiency PCSK1 deficiency LEPR deficiency BBS	hyperpigmentation, nausea, vomiting, and injection site reactions
**Semaglutide**	GLP-1 receptor agonist	2.4 mg	subcutaneous	chronic weight management BMI ≥ 27 kg/m^2^, at least one weight-related ailment or BMI of ≥30 kg/m^2^. Age limit 12 years.	Nausea, vomiting, diarrhea, constipation
**Liraglutide**	GLP-1 receptor agonist	3 mg	subcutaneous	chronic weight management among pediatric patients aged ≥ 12 who are obese	nausea, vomiting, diarrhea, dizziness fever
**Tirzepatide**	GIP receptor and GLP-1 receptor agonist	2.5 mg	subcutaneous	chronic weight management BMI ≥ 27 kg/m^2^, at least one weight-related ailment or BMI of ≥30 kg/m^2^. It is only approved for adults.	nausea, diarrhea, vomiting, constipation, abdominal discomfort and pain, injection site reactions

## Data Availability

No new data were created or analyzed in this study. Data sharing is not applicable to this article.
